# Serum lipid profile in HCV patients treated with direct-acting antivirals: a systematic review and meta-analysis

**DOI:** 10.1038/s41598-021-93251-3

**Published:** 2021-07-06

**Authors:** Rosanna Villani, Francesca Di Cosimo, Antonino Davide Romano, Moris Sangineto, Gaetano Serviddio

**Affiliations:** grid.10796.39 0000000121049995 C.U.R.E. (University Centre for Liver Disease Research and Treatment), Liver Unit, Department of Medical and Surgical Sciences, University of Foggia, Viale Pinto 1, 71122 Foggia, Italy

**Keywords:** Viral hepatitis, Hepatitis C

## Abstract

Although direct-acting antivirals are very effective and safe drugs, several authors have reported the alteration of lipid profile during and after anti-HCV therapy suggesting a potential impact on the risk of cardiovascular events. We performed a systematic review and meta-analysis of observational studies to investigate the magnitude and temporal trend of lipid profile changes in DAA treated patients. All selected studies included data on lipid profile before starting therapy and at least one follow-up assessment during or after antiviral treatment. We identified 14 studies (N = 1537 patients) after removing duplicates. Pooled data showed an increase in total cholesterol 4 weeks after starting therapy (+ 15.86 mg/dl; 95% CI + 9.68 to 22.05; p < 0.001) and 12 weeks after treatment completion (+ 17.05 mg/dl; 95% CI + 11.24 to 22.85; p < 0.001). LDL trend was similar to the total cholesterol change in overall analysis. A mean increase in HDL-cholesterol of 3.36 mg/dl (95% CI + 0.92 to 5.79; p = 0.07) was observed after 12 weeks of treatment, whereas at SVR24 HDL difference was + 4.34 mg/dl (95% CI + 1.40 to 7.28; p = 0.004).Triglycerides did not show significant changes during treatment and after treatment completion. DAAs induce mild lipid changes in chronic hepatitis C patients treated with DAAs, which may persist after treatment completion.

## Introduction

Hepatitis C virus (HCV) infection plays a major role as a cause of chronic liver injury, cirrhosis and hepatocellular carcinoma^1^.

Until 2014, the standard treatment for HCV infection consisted in interferon-based regimens which were associated with unacceptable cure rates and several side effects^2^.

Direct-acting antivirals (DAAs) have revolutionized the treatment of chronic HCV infection because of cure rates approaching 100% with a short duration of treatment^3^.

Eradication of HCV can be reached after only 8–12 weeks of DAA treatment and the benefits impact long-term hepatic and extrahepatic manifestations such as immune-related diseases^4,5^, cardiovascular events^6,7^ and diabetes^8^.

The high efficacy of DAAs has been shown in all patients independently from fibrosis, genotype and age, and it has been associated with an excellent safety profile also in patients ineligible to interferon-based treatment.

In the real clinical setting, several authors have reported changes in serum lipid profile during and after antiviral therapy^2,9–12^. Particularly, DAAs have been suggested to affect low-density lipoprotein (LDL) and, with less consistency, high-density lipoprotein (HDL) and triglyceride serum levels. However, it is unknown whether DAA-induced lipid alterations should be treated, especially in patients affected by cardiovascular disease or metabolic syndrome. Carvalho et al. have recently reported an increased risk of cardiovascular outcomes in HCV patients treated with DAAs due to persistent increase in total cholesterol and LDL^10^.

Since the increase of serum lipids has been reported at different magnitudes in the literature, we addressed the question and performed a systematic review and meta-analysis of observational studies reporting lipid changes during DAA treatment.

Estimating the magnitude and trend of lipid profile may help clinicians to understand to what extent and for how long lipid profile change during DAA treatment.

## Methods

We performed a systematic review and meta-analysis of observational studies according to the criteria of Preferred Reporting Items for Systematic Reviews and Meta-Analyses (PRISMA) statement^13^, Meta‐analysis of Observational Studies in Epidemiology (MOOSE) group^14^ and the Cochrane Handbook for Systematic Reviews^15^.

### Data sources and searches

We searched Pubmed, Web of Science and Scopus for literature published until May 2020.

To identify possible additional studies, we also checked the references of review articles on the topic and bibliography of included articles. We also searched major conference proceedings (American Association for the Study of Liver Disease meeting and European Association for the Study of the Liver International Liver Congress) to identify studies published as abstracts. No publication filters were applied and search results were managed using EndNote X9.

The search strategy was conducted using terms for all classes of oral anti-HCV medications and definite keywords such as “lipid*, cholesterol, LDL, HDL, triglyceride, dyslipid*, hypercholesterolemia, hypertriglyceridemia, metabol*”.

Relevant citations were retrieved after screening of titles and abstracts. The selection of studies based on the inclusion and exclusion criteria was performed independently by two of the authors and conflicts resolved by the third investigator.

### Selection criteria

We selected English-language articles reporting results of observational studies evaluating the safety of antiviral treatment and the effect of DAAs on serum lipid profile.

Eligible studies reported:

- data in adults (> 18 years) with HCV chronic infection treated with DAAs;
- DAA regimen
- data on lipid profile before starting therapy
- at least one follow-up assessment during antiviral treatment and/or after treatment completion

We excluded studies:

1. reporting interferon-containing regimens
2. reporting data on HCV regimens containing non FDA-approved drugs
3. including HIV/HCV patients

### Data extraction and quality assessment

Articles screened by title and abstract were reviewed by two authors who independently performed a full-text analysis for inclusion and exclusion criteria.

They assessed the quality of included studies by using the Newcastle–Ottawa Scale (NOS). The NOS includes three domains: selection, comparability and outcome. It classified the risk of bias as low (7–9 stars; high quality), moderate (4–6 stars; fair quality) and high (1–3 stars; low quality)^16^.

The number of titles/abstracts identified, accepted, and rejected was recorded. Data on key study parameters, including author(s), year of publication, country, sample size, stage of liver fibrosis and antiviral regimens were extracted and recorded in a standardized form.

### Outcomes

The primary outcomes were:

- the magnitude of change in total cholesterol, LDL, HDL and triglyceride during treatment as compared to pre-treatment values (baseline);
- time-course changes during and after DAA treatment completion

To study the temporal trend of serum lipid profile, we performed a timing-based analysis of studies reporting the lipid profile at baseline, during treatment (at week 4, 8, 12 and/or 24 according to treatment duration) and after DAA treatment (12 and/or 24 weeks after treatment completion or longer follow-up when available). SVR12 (Sustained Virological response) and SVR24 are generally defined as aviremia at 12 or 24 weeks, respectively, after completion of therapy.

We also performed subgroup analyses by antiviral regimen and liver fibrosis to assess factors potentially linked to temporal trend and magnitude of lipid changes.

### Statistical analysis

Aggregate study data were used for a quantitative synthesis and the random-effects model of DerSimonian and Laird was used to calculate the mean difference (MD) and 95% confidence intervals (CI) for each time-point. Means and medians were considered equivalent and used directly according to the Cochrane guidelines. Before analysis interquartile ranges were converted to standard deviations (SD) by dividing them by 1.35.

Heterogeneity between studies was evaluated using the I^2^ statistic with a cut-off point of ≥ 50% and a p value < 0.10 on the χ^2^ test was defined as a significant degree of heterogeneity.

Potential sources of heterogeneity were studied by subgroup analyses according to study characteristics.

Publication bias was explored quantitatively performing the Egger’s test. Funnel plot and trim and fill analysis were performed when more than 10 studies were available for each time point according to the Cochrane Handbook.

Statistical analyses were performed using STATA (version 14; STATA Corporation, College Station, TX).

## Results

### Characteristics and quality of included studies

Study characteristics and the PRISMA flow chart are shown in Table 1 and Supplementary Fig. S1, respectively.

**Table 1:** Characteristics of included studies.

Author	Country	Antiviral regimens	Patients (N)	Male (N)	Age (years)	Genotype	Weeks of treatment	Fibrosis	Cirrhosis	Patients taking lipid-lowering drugs % (N)
Endo et al 2017 WJG	Japan	DCV/ASV (Group1)	121	59	68.4 ± 11.8	G1b	24	F0–F4	n.a	1.6% (2)
		SOF/LDV (Group 2)	132	49	66.7 ± 13.1	G1b	24	F0–F4	n.a	0
Chida et al 2018 Gut Liver	Japan	DCV/ASV	70	28	71 ± 9	G1b	24	n.a	n.a	Excluded
Inoue et al 2018 Hepatol Res	Japan	DCV/ASV (Group 1)	85	33	68.3 ± 10.5	G1b	24	F0–F4	n.a	n.a
		SOF/LDV (Group 2)	85	37	64.4 ± 13.3	G1b	12	F0–F4	n.a	n.a
		SOF/RBV (Group 3)	46	15	62.0 ± 15.3	G2	12	F0–F4	n.a	n.a
Sun et al 2018 Gut	Taiwan	GZR/EBV or SOF/LDV	24	12	60 (39–83)	G1	12	F0–F4	n.a	n.a
El Sagheer et al 2018 Lybian J Med	Egypt	SOF/SMV	80	47	47 ± 12	G4	12	F0–F4	35%	excluded
Shimizu et al 2018 Sci Rep	Japan	Multiple DAA regimens	70	29	66 (59–73)	G1/G2	12/24	F0–F4	n.a	n.a
Gitto et al 2018 Ann Hepatol	Italy	Multiple DAA regimens	100	59	47 ± 12	Multiple genotypes	12/24	F1–F4	81%	2% (2)
Cheng et al 2019 Sci Rep	Taiwan	Multiple DAA regimens	102	34	66.0 ± 10.7	Multiple genotypes	12/24	F0–F4	74%	19% (21)
Jain et al 2019 Indian J Gastroenterol	India	SOF + DCV	50	30	38 ± 13	G3	12	F0–F4	n.a	excluded
Petta et al 2018 J Hepatol	Italy	Multiple DAA regimens	182	102	63.1 ± 10.4	Multiple genotypes	12/24	F3–F4	65.9%	excluded
Ichikawa et al 2019 Biomed Rep	Japan	DCV/ASV	39	14	70.9 ± 11	G1b	24	n.a	35.9%	5.1% (2)
Hashimoto et al 2016 Plos One	Japan	SOF/LDV (Group 1)	76	31	69.0 (63.0, 75.0) iqr	G1	12	F0–F4	67.1%	excluded
		DCV/ASV (Group 2)	24	8	76.0 (69.0, 80.0)iqr		24	F0–F4	79.2%	
Doyle et al 2019 Cells	Canada	PrOD	24	17	54 ± 11.6	G1	12	F0–F4	24%	excluded
Juanbeltz et al 2019 Postgrad Med	Spain	Multiple DAA regimens	227	163	53.6 (9.3)	Multiple genotypes	12/16/24	F0–F4	40.5%	excluded

Starting from 1983 citations, we identified 14 studies including 1537 patients with serial assessment of lipid profile.

Fourteen studies included data on total cholesterol^2,12,17–28^; eleven studies reported data on HDL-cholesterol^2,12,17,19,20,22–26,28^; temporal trend on LDL-cholesterol was reported by twelve studies^2,12,17–20,22–26,28^ and, finally, triglyceride profile on treatment or after antiviral therapy was described by ten authors^12,20–26,28^. Nine studies were conducted in Asia (Japan, Taiwan and India), three in Europe, one in Africa and one in North America.

Seven studies excluded patients taking statins, three studies did not report data on concomitant lipid-lowering drugs, whereas four studies included a not significant number of patients taking medications to treat lipid disorders.

The largest part of the selected studies included patients with all stages of fibrosis, whereas one study^27^ included only patients with F3 and F4 stages of liver fibrosis.

The Egger regression test for the evaluation of publication bias did not show small study effects (p = 0.23). The quality assessment scores are shown in Supplementary Table S1. All included studies had low or moderate risk of bias.

### Change in total cholesterol and LDL-cholesterol during and after antiviral therapy

**Figure 1:**
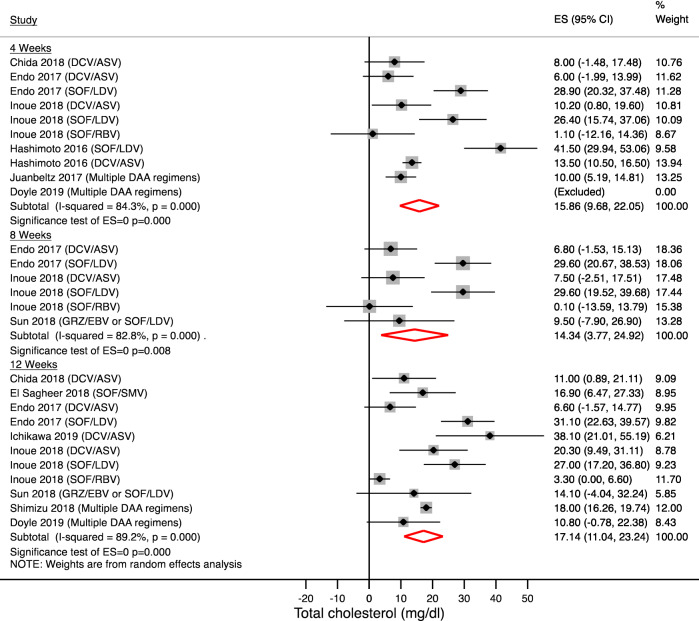
Serum total cholesterol changes during treatment. Forest plot for mean difference (MD) and 95% confidence intervals (CI) for each time-point. *DCV/ASV* daclatasvir/asunaprevir, *SOF/LDV* sofosbuvir/ledipasvir, *SOF/RBV* sofosbuvir + ribavirin, *SOF/SMV* sofosbuvir/simeprevir, *GRZ/EBV* grazoprevir/elbasvir.

**Figure 2:**
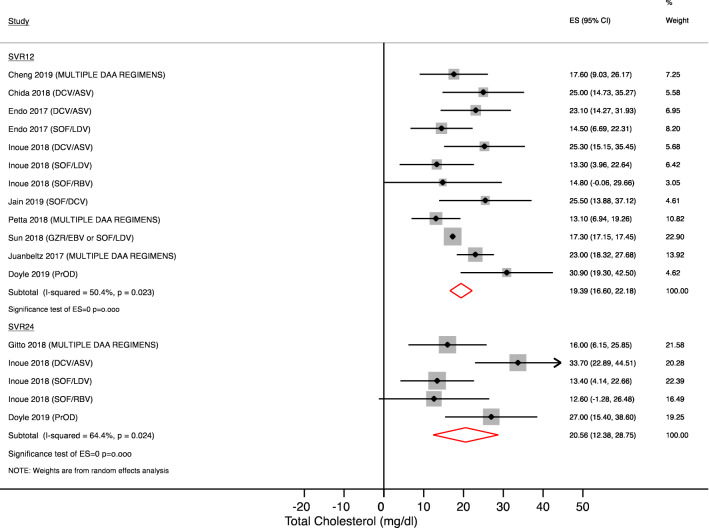
Serum total cholesterol changes after treatment completion. Forest plot for mean difference (MD) and 95% confidence intervals (CI) for each time-point. *DCV/ASV* daclatasvir/asunaprevir, *SOF/LDV* sofosbuvir/ledipasvir, *SOF/RBV* sofosbuvir + ribavirin, *SOF/SMV* sofosbuvir/simeprevir, *GRZ/EBV* grazoprevir/elbasvir, *PrOD* paritaprevir/ritonavir/ombitasvir + dasabuvir, *SVR12* Sustained virological response at 12 weeks; SVR24: Sustained virological response at 24 weeks.

Figures 1 and 2 show the magnitude and kinetics of serum total cholesterol changes during and after treatment. Serum concentration of total cholesterol significantly increased 4 weeks after starting therapy by 15.86 mg/dl (95% CI + 9.68 to 22.05; N = 940 patients; p < 0.001) and remained higher at week 8 (+ 14.34 mg/dl [95% CI + 3.77 to 24.92]; N = 493 patients; p = 0.008) and after 12 weeks of treatment (17.14 mg/dl (95% CI + 11.04 to 23.24; N = 776 patients; p < 0.001). These changes persisted after 12 weeks (+ 19.39 mg/dl [95% CI + 16.60 to 22.18]; N = 1148 patients; p < 0.001) and 24 weeks (+ 20.56 mg/dl [95% CI + 12.38 to 28.75]; N = 340 patients; p < 0.001) from treatment completion.

Because of high heterogeneity, we analyzed the results after stratification by treatment as reported in Table 2. Only 2 antiviral regimens were available for subgroup analysis (daclatasvir/asunaprevir and sofosbuvir/ledipasvir) and results showed that sofosbuvir/ledipasvir use was associated with a higher total cholesterol increase on treatment. On the contrary daclatasvir/asunaprevir use showed lower total cholesterol increase compared to daclatasvir/asunaprevir therapy on treatment, whereas a more significant increase occurred after treatment completion.

**Table 2:** Serum lipid changes by antiviral regimen.

Subgroup by DAA regimen	Studies (N) and references	4 Weeks MD (95% CI) mg/dl	Studies (N) and references	12 Weeks MD (95% CI) mg/dl	Studies (N) and references	SVR12 MD (95% CI) mg/dl
**Total cholesterol**						
DCV/ASV	4^2,12,17,18^	+ 11.05 (7.36–14.74) p < 0.001 I^2^ = 24.1% (p = 0.267)	4^2,12,17,23^	+ 17.25 (6.09–28.42) p = 0.002 I^2^ = 75.9% (p = 0.006)	3^2,12,17^	+ 24.33 (18.74–29.91) p < 0.001 I^2^ = 0% (p = 0.939)
SOF/LDV	3^2,12,18^	+ 31.79 (23.35–40.22) p < 0.001 I^2^ = 51.3% (p = 0.128)	3^2,12,21^	+ 26.98 (19.46–34.49) p < 0.001 I^2^ = 28.5% (p = 0.247)	3^2,12,21^	+ 17.29 (17.14–17.44) p < 0.001 I^2^ = 0% (p = 0.549)
**LDL cholesterol**						
DCV/ASV	4^2,12,17,18^	+ 9.68 (5.39–13.97) p < 0.001 I^2^ = 46.7% (p = 0.131)	3^2,12,17^	+ 6.57 (1.95–11.18) p = 0.005 I^2^ = 0% (p = 0.688)	4^2,12,17,23^	+ 20.92 (16.31–25.53) p < 0.001 I^2^ = 0% (p = 0.708)
SOF/LDV	3^2,12,18^	+ 25.52 (19.33–31.71) p < 0.001 I^2^ = 35.3% (p = 0.213)	2^2,12^	+ 21.57 (16.11–27.04) p < 0.001 I^2^ = 0% (p = 0.793)	2^2,12^	+ 9.28 (4.34–14.21) p < 0.001 I^2^ = 0% (p = 0.586)
**HDL cholesterol**						
DCV/ASV	3^2,12,17^	− 3.10 (− 7.294 to + 1.094) p = 0.630 I^2^ = 64.4% (p = 0.06)	3^2,12,17^	+ 2.34 (− 1.611 to 6.31) p = 0.245 I^2^ = 53.2% (p = 0.118)	4^2,12,17,23^	+ 5.08 (2.44–7.71) p < 0.001 I^2^ = 0% (p = 0.628)
SOF/LDV	2^12^	+ 5.72 (2.99–8.44) p < 0.001 I^2^ = 0% (p = 0.944)	2^2,12^	+ 7.51 (3.90–11.13) ﻿p < 0.001 I^2^ = 14.9% (p = 0.27)	2^2,12^	+ 3.08 (− 0.82 to + 7.00) p = 0.122 I^2^ = 30% (p = 0.632)

*DCV/ASV* daclatasvir/asunaprevir, *SOF/LDV* sofosbuvir/ledipasvir, *MD* mean difference, *SVR12* sustained virological response at 12 week.

**Figure 3:**
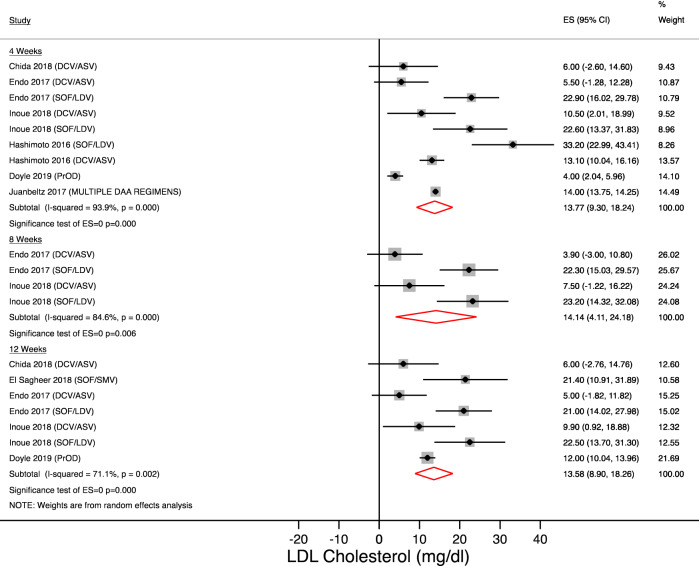
LDL-cholesterol changes during treatment. Forest plot for mean difference (MD) and 95% confidence intervals (CI) for each time-point. *DCV/ASV* daclatasvir/asunaprevir, *SOF/LDV* sofosbuvir/ledipasvir, *SOF/RBV* sofosbuvir + ribavirin, *SOF/SMV* sofosbuvir/simeprevir, *GRZ/EBV* grazoprevir/elbasvir.

**Figure 4:**
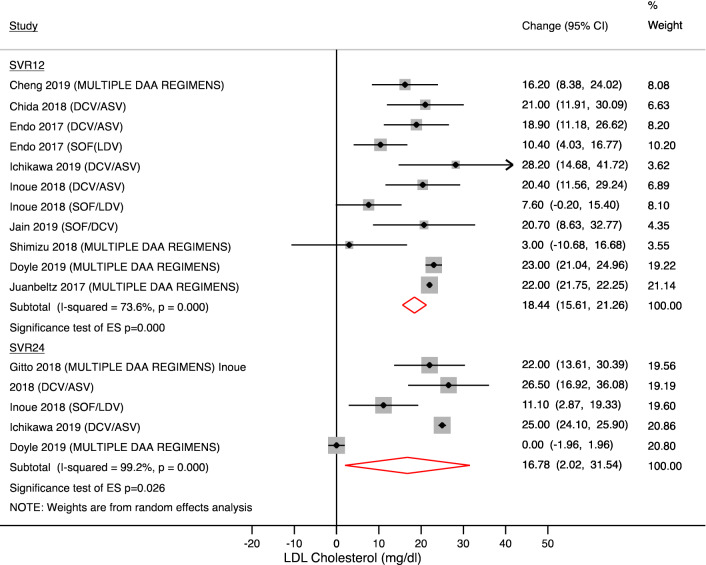
LDL-cholesterol changes after treatment completion. Forest plot for mean difference (MD) and 95% confidence intervals (CI) for each time-point. *DCV/ASV* daclatasvir/asunaprevir, *SOF/LDV* sofosbuvir/ledipasvir, *SOF/RBV* sofosbuvir + ribavirin, *SOF/DCV* sofosbuvir + daclatasvir, *SVR12* Sustained virological response at 12 weeks, *SVR24* Sustained virological response at 24 weeks.

The magnitude and temporal trend of LDL cholesterol is showed in Figs. 3 and 4. Changes in LDL cholesterol were similar to total cholesterol values. After 4 weeks of treatment, LDL cholesterol increased by 13.77 mg/dl (95% CI + 9.30 to 18.24; N = 844 patients; p < 0.001) and comparable findings were observed after 8 weeks (14.14 mg/dl [95% CI + 4.11 to 24.18]; N = 423 patients; p = 0.006) and 12 weeks of treatment (13.58 mg/dl [95% CI + 8.9 to 18.26] N = 597 patients; p < 0.001).

After treatment completion, the increase in LDL cholesterol was confirmed at SVR12 and SVR24 (+ 18.44 mg/dl [95% CI + 15.61 to 21.26] and + 16.78 mg/dl [95% CI + 2.02 to 31.54], respectively).

Subgroup analysis reported in Table 2 showed a magnitude and temporal trend, which is concordant with serum total cholesterol changes.

### Change in HDL cholesterol

**Figure 5:**
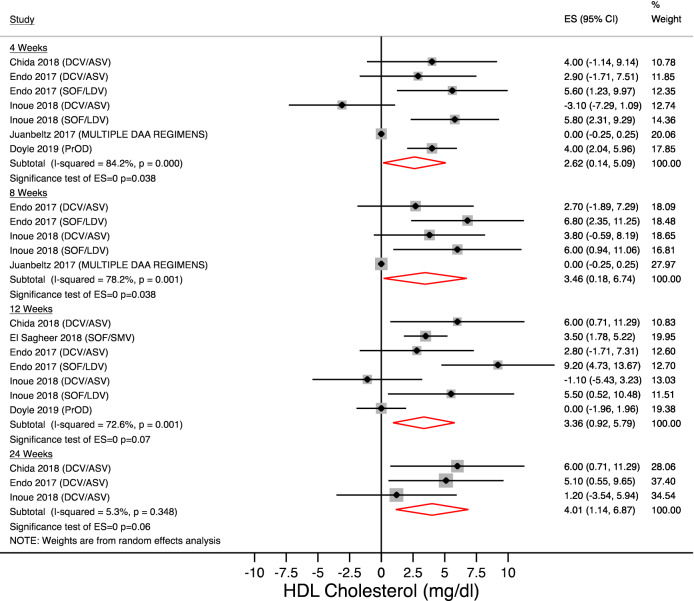
HDL-changes during treatment. Forest plot for mean difference (MD) and 95% confidence intervals (CI) for each time-point. *DCV/ASV* daclatasvir/asunaprevir, *SOF/LDV* sofosbuvir/ledipasvir, *SOF/RBV* sofosbuvir + ribavirin, *SOF/SMV* sofosbuvir + simeprevir.

**Figure 6:**
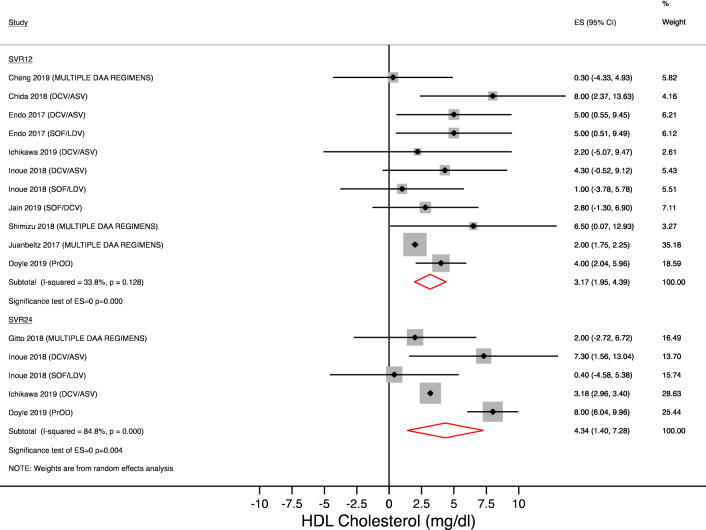
HDL-changes after treatment completion. Forest plot for mean difference (MD) and 95% confidence intervals (CI) for each time-point. *DCV/ASV* daclatasvir/asunaprevir, *SOF/LDV* sofosbuvir/ledipasvir, *PrOD* paritaprevir/ritonavir/ombitasvir + dasabuvir, *SOF/SMV* sofosbuvir + simeprevir.

Figures 5 and 6 show the change in HDL-cholesterol on treatment and after treatment completion.

Our analysis showed a mean increase in HDL-cholesterol between 2.62 mg/dl and 4 mg/dl on treatment and between 3.17 mg/dl and 4.34 mg/dl after the end of treatment. In the overall analysis, these findings were statistically significant at 4 and 8 weeks and at SVR12 and SVR24. Heterogeneity was significant at each time point (Figs. 5 and 6), therefore we performed a subgroup analysis (Table 2).

Different results were observed by antiviral regimens; particularly patients taking sofosbuvir/ledipasvir developed an increase in HDL cholesterol on treatment (at 12 week : + 7.51 mg/dl [95% CI 3.90–11.13; p < 0.001]) that disappeared at SVR12 (+ 3.08 mg/dl [95% CI − 0.82 to + 7.00; p = 0.122]). On the contrary, patients taking daclatasvir/asunaprevir did not show significant changes in HDL cholesterol during treatment (at 12 week: + 2.34 mg/dl [95% CI − 1.611 to + 6.31; p = 0.245]) whereas statistically significant findings occurred at SVR12 (+ 5.08 mg/dl [95% CI 2.44–7.71; p = 0.000]).

### Change in triglyceride level during and after antiviral therapy

**Figure 7:**
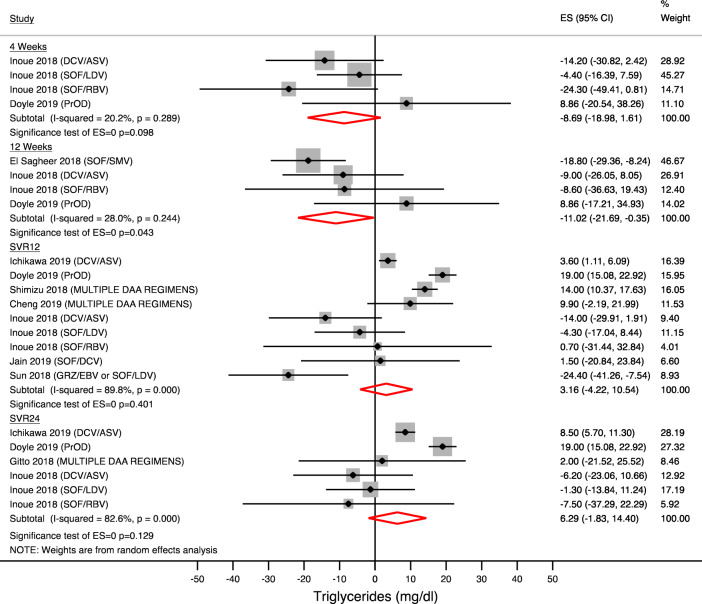
Serum Triglycerides changes during and after treatment. Forest plot for mean difference (MD) and 95% confidence intervals (CI) for each time-point. *DCV/ASV* daclatasvir/asunaprevir, *SOF/LDV* sofosbuvir/ledipasvir, *PrOD* paritaprevir/ritonavir/ombitasvir + dasabuvir, *SOF/DCV* sofosbuvir + daclatasvir, *SVR12* Sustained virological response at 12 weeks, *SVR24* Sustained virological response at 24 weeks.

Ten studies reported data on temporal trends of triglycerides during and after HCV treatment. Figure 7 shows the pooled data, which did not reveal any changes on treatment (at 4 week − 8.69 mg/dl; 95% CI − 18.98 to + 1.61; p = 0.098) and after treatment completion (SVR12 3.16 mg/dl; 95% CI − 4.22 to + 10.54; N = 525 patients; p = 0.401). Subgroup analysis by antiviral regimens was not performed because of different antiviral treatment in studies included in the final analysis.

### Lipid profile in liver cirrhosis

All studies included in our analysis did not report lipid changes in subgroups by liver fibrosis however some of them included a large number of cirrhotic patients. They are discussed as follows.

Cheng et al. compared lipid profiles in patients with liver stiffness ≥ 9.5 kPa (N = 76 patients) versus patients with mild fibrosis (N = 26)^25^. The difference in serum cholesterol, LDL and HDL between baseline and SVR12 was statistically significant both in patients with non advanced-fibrosis and in cirrhotics whereas triglyceride levels showed a significant increase only in patients without advanced fibrosis.

Petta et al. included only patients with significant fibrosis (N = 182) and more than 65% of them had liver cirrhosis. The authors did not report results according to liver fibrosis stage; however, total cholesterol increase at SVR12 was comparable to data (+ 13.10 mg/dl [95% CI + 6.94 to + 19.26]) reported by other authors. Results from an Italian cohort by Gitto et al.^28^ including a very large proportion of cirrhotics (81% of study population; N = 100 patients) showed that total cholesterol and LDL-cholesterol were significantly increased at SVR24 whereas triglyceride and HDL-cholesterol serum levels were not changed on treatment and after treatment completion.

Finally, Hashimoto et al. reported data from a population including about 80% of cirrhotic patients.

After only 4 weeks of treatment, total cholesterol and LDL cholesterol showed a significant increase whereas triglycerides were not significantly modified by antiHCV treatment.

## Discussion

With the advent of the direct-acting antiviral agents, the long-established goal of eradicating HCV has been achieved with very effective and safe drugs.

It has been recently suggested that DAA-induced viral clearance may produce metabolic benefits such as steatosis improvement and atherosclerosis reduction^6,24,29–31^. These beneficial effects are probably related to the removal of negative impact of HCV on lipogenesis and pro-inflammatory cytokine release and they presumably account for the cardiovascular benefits of HCV cure.

At the same time, some authors have reported the worsening of lipid profile during DAA treatment raising the issue of potential negative consequences of antiHCV therapy in patients with previous cardiovascular disease.

Carvalho et al*.* observed a significant increase in LDL, total cholesterol and HOMA in patients who achieved SVR after DAA treatment and speculate on potential negative effects of interferon-free therapy on cardiovascular risk^10^. Gitto et al. found pro-atherogenic lipid changes during treatment associated with improvements of insulin resistance. They raised the issue of global cardiovascular balance in patients with amelioration of glucose metabolism and concurrent negative changes of lipid profile^28^.

Our meta-analysis summarizes data from fourteen observational studies on the magnitude of serum lipid profile changes during DAA therapy.

Our results showed that DAA treatment has a mild but significant effect on serum cholesterol during treatment irrespective of antiviral regimens. The increase is mainly dependent on LDL change observed immediately after starting treatment.

In the overall analysis, we observed an average increase of total cholesterol of 19 mg/dl at SVR12 and 20 mg/dl at SVR24. From a clinical point of view, these findings seem not be relevant for the cardiovascular risk. However, subgroups analysis by antiHCV regimen revealed very challenging results. Antiviral regimens including a protease inhibitor showed a lower increase in total cholesterol at 4 weeks and a progressive increase with a significant worsening of total cholesterol serum level at SVR12 (+ 24 mg/dl) whereas antiHCV treatment based on protease-inhibitor free regimens showed a considerable and early increase in total cholesterol (+ 31 mg/dl at 4 week) with a less significant increase at SVR12.

Long-term DAA effects are reported by only 2 authors who studied lipid profiles after 1 year from the start of the therapy.

Carvalho et al. studied 178 patients who achieved SVR and confirmed the increase in total cholesterol and LDL cholesterol after one year of follow-up. Similarly, Ichikawa et al. showed that total and LDL cholesterol were significantly increased 36 weeks and 52 weeks after the start of treatment in patients who received daclatasvir and asunaprevir for 24 weeks.

Further long-term studies are needed to confirm these preliminary data which suggest a potential durable effect of AntiHCV treatment on the lipid profile.

Extensive literature data have shown that chronic HCV infection is involved in significant alteration of lipid profile^32^ because HCV stimulates LDL receptor expression in hepatic cells. It also negatively modulates the expression of PCSK9 (Proprotein Convertase Subtilin/Kexin type 9) that promotes LDL receptor degradation^33^.

Moreover, HCV modulates the activity of microsomal triglyceride transfer protein (MTP) and sterol-regulatory-element-binding-protein (SREBP) involved in the pathogenesis of liver steatosis and hypolipidemia^32^.

In agreement with these data, the eradication of HCV infection with IFN-based regimens has shown normalization of hypolipidemia^34^ and a significant improvement of liver steatosis in patients with SVR^35^.

After DAA approval, several authors have observed lipid metabolism disorders during treatment suggesting that several factors are involved in their pathogenesis. Most manuscripts have reported a significant increase in total and LDL cholesterol as previously discussed however data on triglycerides and HDL had different magnitude and temporal trends.

Our meta-analysis showed that HDL had a positive trend, which is statistically significant however it seems not to be interesting from a clinical perspective.

Indeed, the HDL cholesterol showed a mean increase of 3 mg/dl (95% IC 1.95–4.39) at SVR12 and similar results were observed on treatment.

As well, our results showed a not significant impact of DAAs on serum triglyceride level on treatment and after treatment completion.

Our study is the first systematic review and meta-analysis, which analyzes the magnitude and kinetics of lipid changes during and after DAA treatment by a complete and updated revision of the literature on the topic.

Limitations were the small number of studies included in the statistical analysis, the inclusion of observational studies, the potential bias from selecting only English-language studies, the lack of very long-term follow-up data, the limited geographical origin of population and finally the lack of stratification by fibrosis.

However, we demonstrated that DAAs can induce lipid changes in patients with chronic hepatitis C which may persist after treatment completion. These include a significant increase in LDL, HDL and total cholesterol, but no changes in triglycerides.

Even if statistically significant, the magnitude of changes improbably may account for clinical implication in terms of cardiovascular risk.

Therefore, future studies should be designed to estimate the cardiovascular impact of these changes in high risk CHC patients, and to assess the clinical benefits associated with the use of lipid lowering agents.

## Supplementary Information

Supplementary Information.

## Data Availability

All data generated or analysed during this study are included in this published article (and its Supplementary Information files).
